# Characterization of the *Candida orthopsilosis* agglutinin-like sequence (*ALS*) genes

**DOI:** 10.1371/journal.pone.0215912

**Published:** 2019-04-24

**Authors:** Lisa Lombardi, Marina Zoppo, Cosmeri Rizzato, Daria Bottai, Alvaro G. Hernandez, Lois L. Hoyer, Arianna Tavanti

**Affiliations:** 1 Department of Biology, University of Pisa, Pisa, Italy; 2 Roy J. Carver Biotechnology Center, University of Illinois at Urbana-Champaign, Urbana, Illinois, United States of America; 3 Department of Pathobiology, University of Illinois at Urbana-Champaign, Urbana, Illinois, United States of America; Institut de Genetique et Microbiologie, FRANCE

## Abstract

Agglutinin like sequence (Als) cell-wall proteins play a key role in adhesion and virulence of *Candida* species. Compared to the well-characterized *Candida albicans ALS* genes, little is known about *ALS* genes in the *Candida parapsilosis* species complex. Three incomplete *ALS* genes were identified in the genome sequence for *Candida orthopsilosis* strain 90–125 (GenBank assembly ASM31587v1): *CORT0C04210* (named *CoALS4210*), *CORT0C04220* (*CoALS4220*) and *CORT0B00800* (*CoALS800*). To complete the gene sequences, new data were derived from strain 90–125 using Illumina (short-read) and Oxford Nanopore (long-read) methods. Long-read sequencing analysis confirmed the presence of 3 *ALS* genes in *C*. *orthopsilosis* 90–125 and resolved the gaps located in repetitive regions of *CoALS800* and *CoALS4220*. In the new genome assembly (GenBank PQBP00000000), the *CoALS4210* sequence was slightly longer than in the original assembly. *C*. *orthopsilosis* Als proteins encoded features well-known in *C*. *albicans* Als proteins such as a secretory signal peptide, N-terminal domain with a peptide-binding cavity, amyloid-forming region, repeated sequences, and a C-terminal site for glycosylphosphatidylinositol anchor addition that, in yeast, suggest localization of the proteins in the cell wall. CoAls4210 and CoAls800 lacked the classic *C*. *albicans* Als tandem repeats, instead featuring short, imperfect repeats with consensus motifs such as SSSEPP and GSGN. Quantitative RT-PCR showed differential regulation of *CoALS* genes by growth stage in six genetically diverse *C*. *orthopsilosis* clinical isolates, which also exhibited length variation in the *ALS* alleles, and strain-specific gene expression patterns. Overall, long-read DNA sequencing methodology was instrumental in generating an accurate assembly of *CoALS* genes, thus revealing their unconventional features and first insights into their allelic variability within *C*. *orthopsilosis* clinical isolates.

## Introduction

Fungal adhesion is essential for stable colonization of host surfaces and subsequent disease development. Adhesion is mediated by molecules exposed on the fungal cells and accessible ligands on the host surface. Fungal adhesins include glycosylphosphatidylinositol (GPI)-modified cell wall proteins that mediate interactions with host cells, resident microbiota, and abiotic surfaces (reviewed in de Groot et al., [1]).

Proteins in the Agglutinin-Like Sequence (*ALS*) family of *Candida albicans* are among the best-characterized fungal adhesins [2]. In this species, the *ALS* family includes eight genes that encode large, cell-surface glycoproteins that share a similar basic organization including an N-terminal domain with adhesive function (NT-Als), a central domain of tandemly repeated sequences, and a Ser/Thr-rich C-terminal domain. The presence of a secretory signal sequence at the N terminus of the protein and a GPI anchor addition site at the C terminus are consistent with protein entry into the secretory pathway, and final localization linked to β-1,6-glucan in the fungal cell wall [3].

The molecular basis for adhesive function was first described for *C*. *albicans* Als9 by solving its NT-Als structure [4]. The NT-Als structure shows two immunoglobulin-like domains that form a peptide-binding cavity that can contain up to 6 amino acids. The flexible C-terminal ends of peptide ligands make natural binding partners due to an invariant Lys at the bottom of the binding cavity that can form an ionic pair with the C-terminal carboxyl group of the incoming peptide. Further functional characterization was pursued using *C*. *albicans* Als3 because it makes the largest contribution to *C*. *albicans* adhesion to host cells (reviewed in [5] and [6]). Mutagenesis of key amino acids within the peptide-binding cavity did not alter NT-Als surface topography. When the mutant construct was introduced into *C*. *albicans* under control of the *ALS3* promoter, the resulting strain produced Als3 on the cell surface in quantities similar to wild-type, but had the adhesive capacity of a *Δals3/Δals3* null mutant that had no surface Als3 [7]. This work demonstrated the importance of the peptide-binding cavity in Als-mediated adhesion. Als proteins also have a short sequence with amyloid-forming potential [8] that contributes to the aggregative properties of the protein. Overall, these features promote *C*. *albicans* interaction with complex surfaces including host cells, other microbes, and protein-coated abiotic materials [7].

Despite the clinical relevance of species in the *Candida parapsilosis sensu latu* complex [9, 10], including *C*. *parapsilosis*, *Candida orthopsilosis*, and *Candida metapsilosis*, little is known about the Als proteins in these species. Previous data indicate that *C*. *parapsilosis* and *C*. *orthopsilosis* have comparable adhesive properties, while *C*. *metapsilosis* is the least adhesive species of the complex [11]. Over the past few years, the availability of genome sequences and annotations for *C*. *parapsilosis* and *C*. *orthopsilosis* led to identification of *ALS*-like genes in these opportunistic pathogens ([12], [13]). The *ALS* gene composition of *C*. *parapsilosis* is variable depending on the strain examined [14]. Strain CDC 317, for which the annotation is featured in the *Candida* Genome Database (http://www.candidagenome.org), has five *ALS* genes whereas other *C*. *parapsilosis* strains have one or three. Disruption of *CPAR2_404800* reduced *C*. *parapsilosis* adhesion to human buccal epithelial cells [15], suggesting that *C*. *parapsilosis* Als proteins function in adhesion like their *C*. *albicans* orthologs. The goal of this work was to identify and characterize the *ALS* gene family of *C*. *orthopsilosis* using genome sequence data. Because of the presence of extensive tracts of repeated sequences, *ALS* genes are often incomplete in genome assemblies. Here, we describe the process of completing the sequences of the *C*. *orthopsilsosis ALS* genes and their encoded proteins. A set of *C*. *orthopsilosis* clinical isolates was used to examine sequence variation and relative *ALS* gene expression patterns. The resulting data provide insight into the *ALS* gene family in *C*. *orthopsilosis* and the basis for functional characterization.

## Materials and methods

### Fungal strains and growth conditions

The *C*. *orthopsilosis* type strain ATCC 96139 [16] and the genome sequencing strain 90–125 [13] were included in this study, along with 4 clinical isolates (124, 85, 331, and 488) that were part of a strain collection deposited at the Department of Biology, University of Pisa. *C*. *orthopsilosis* strains were maintained as 30% glycerol frozen stocks at -20°C or -80°C and cultured on YPD agar plates (per liter: 10 g yeast extract, 20 g peptone, 20 g dextrose, 15 g agar). YPD liquid medium was used for routine growth at 30°C with shaking.

### Genomic DNA preparation

*C*. *orthopsilosis* genomic DNA for PCR amplification was extracted after an overnight incubation at 30°C in YPD medium with shaking. Cells were resuspended in a lysis buffer, broken with glass beads, and the resulting suspension extracted with phenol:chloroform:isoamyl alcohol (25:24:1) as described previously [16]. Following RNase treatment, DNA was precipitated with 2 volumes of isopropanol and 10 μl of 4 M ammonium acetate. The pellet was dried and dissolved in 50 μl of TE (pH 8.0).

*C*. *orthopsilosis* genomic DNA for long-read sequencing was extracted from cells that were grown for 16 h at 37°C in YPD medium with 200 rpm shaking. Cells were treated with zymolyase to form spheroplasts that were lysed with sodium dodecyl sulfate. Gentle mixing by inversion was used to handle the spheroplasts, and during phenol extractions and isopropyl alcohol precipitation of DNA [17].

### Genome sequence data generation and assembly

New genome data were derived from strain 90–125 using Illumina (short-read) and Oxford Nanopore (long-read) methods. MiSeq shotgun genomic libraries were prepared with the Hyper Library construction kit (Kapa Biosystems). The library was quantitated by qPCR and sequenced on one MiSeq flowcell for 151 cycles from each end of the fragment using a MiSeq 300-cycle sequencing kit (version 2). FASTQ files were generated and demultiplexed with the bcl2fastq Conversion Software (Illumina, version 2.17.1.14). MiSeq reads were quality trimmed using Trimmomatic [18] with the parameters “LEADING:30 TRAILING:30” prior to assembly. MiSeq yielded 2,281,330 reads of 150 nt each.

For Oxford Nanopore long-read sequencing, 1 μg of genomic DNA was sheared in a gTube (Covaris, Woburn, MA) for 1 min at 6,000 rpm in a MiniSpin plus microcentrifuge (Eppendorf, Hauppauge, NY). The sheared DNA was converted into a Nanopore library with the Nanopore Sequencing kit (LSK-108) with the Expansion barcoding kit (EXP-NBD103; Oxford Nanopore, UK). The library was sequenced on a SpotONFlowcell MK I (R9.4) for 48 h using a MinION MK 1B sequencer. Base calling and demultiplexing were performed in real time with the Metrichor Agent V2.45.3 using the 1D Base Calling plus Barcoding for FLO-MIN_106D 450 bp workflow. Sixty nucleotides (nt) were removed from both ends of each Oxford Nanopore read. Reads longer than 1000nt were used in the final assembly. The Oxford Nanopore (ONP) flow cell yielded 40,744 reads for a total of 364,246,709 bp. The mean and median ONP read lengths were 8,940 and 8,754 bp, respectively with a minimum of 114 bp and a maximum of 98,108 bp.

Genome assembly was performed using Canu v1.4 [19] using default parameters with the command ‘canu -p asm -d orthopsilosisgenomeSize = 14m useGrid = false -nanopore-raw C_orthopsilosis_trimmed.fastq’ using the trimmed ONP FASTQ reads. ONP reads were then aligned against the assembly using bwa [20], and the alignment was then used to polish the assembly using nanopolish v 0.6.0 [21]. Finally, the trimmed MiSeq data was used to additionally polish the assembly using Pilon v1.21 [22].

### Identification and *in silico* analysis of *ALS* genes

*C*. *orthopsilosis ALS* genes were identified by command-line BLAST of the entire genome sequence using known *ALS* sequences as the query. *C*. *albicans ALS3* (GenBank accession number AY223552) provided a baseline query because of its prototypical N-terminal (NT-Als) domain sequence for which the three-dimensional structure is known [7], as well as the central tandem-repeat domain (TR; head-to-tail copies of a 36-amino acid repeated sequence) and a Ser/Thr-rich C-terminal (CT) domain present in the translated protein. Other query sequences included *C*. *albicans ALS1* (L25902), *ALS2* (AH006927), *ALS4* (AH006929), *ALS5* (AY227440), *ALS6* (AY225310), *ALS7* (AF201684), *ALS9-1* (AY269423), and *ALS9-2* (AY269422). Once identified, *CoALS* genes were used as BLAST queries, as well. The putative cleavage site of the N-terminal signal peptide was predicted using SignalP 4.1 Server (http://www.cbs.dtu.dk/services/SignalP; [23]). Tango *in silico* analysis (http://tango.crg.es; [24]) was used to identify the amyloid-forming region (AFR). The hypothetical position of the ω site to which the GPI moiety is attached after proteolytic cleavage was predicted by using PredGPI (http://gpcr.biocomp.unibo.it/predgpi/index.htm; [25]). Tandem repeat units were detected with T-Reks (http://bioinfo.montp.cnrs.fr/?r=t-reks; [26]).

### Analysis of *ALS* allelic variation

PCR was used to amplify various *CoALS* fragments to detect allelic size variation. Primers were designed according to the genomic sequence of the strain 90–125 available in the *Candida* Gene Order Browser database (CGOB3, http://cgob3.ucd.ie; [27, 28]; Table 1). PCRs used DreamTaq DNA Polymerase (Thermo Fisher Scientific); primers were synthesized by Sigma Genosys or Integrated DNA Technologies. Amplification of entire *CoALS* genes used Q5 High-Fidelity DNA polymerase (New England Biolabs, NEB). PCRs were heated at 98°C for 30 s followed by 30 cycles of 98°C (10 s), 68°C (30 s), and 72°C (2.5 min for *ALS800* and *ALS4210*, and 3.5 min for *ALS4220*). A final 10-min extension 72°C was performed. PCR products were migrated on a 0.8% agarose gel in Tris Acetate EDTA buffer (TAE). Molecular sizes were calculated *in silico* using Gel Analyzer 2010 software (http://www.gelanalyzer.com/index.html) and either the GeneRuler 1 kb DNA ladder (Thermo Fisher Scientific) or 100 bp DNA ladder (NEB).

**Table 1:** Oligonucleotide primers used in this work.

Primer	Sequence (5’-3’)	Description
CO800EF2	AAGACACGCGGGAACAATCT	Amplification of *CoALS* genes
CO800ER2	GGGCGGCAATTCTGATGTTG	
CO4210EF2	ACCACAGTGCTCCACAACAA	
CO4210ER2	CCTTCGCTCCCAAACAGGC	
CO4220EF1	ACGCTCAGCCCAATAACCAA	
CO4220ER1	ATCCTTGGCTGCTGATGCTT	
Seq800F1	GTAGGGCTCAGTTAAGTTC	Sequencing of N-terminal domain
Seq800R1	CCAAATCACCAGGAGCAAAC	
Seq4210F1	CGAGATGCAAAACTCCTATCCC	
Seq4210R1	CACCAGGCGTAGCTGTGATA	
Seq4220F1	CCCAGTAGGCGAACTAAATG	
Seq4220R1	TTAGTGTTGGAACCACCTTG	
800ThrFWD^a^	CCTAACGATGGATCAAGATC	Evaluation of *CoALS800* allelic variability ^a,b,c^ indicate primer pairs used for PCR analysis represented in Fig 4 ^a^*^,b^*^,c^* indicate primer pairs used for PCR analysis represented in Fig 5
800ThrREV^a^	AGTAAGAGTTATCGGATCCC	
800StalkFWD^b,a^*	GGGATCCGATAACTCTTACT	
800StalkREV^b,c^*	TATTACGGTGCTGGTAACTG	
LL7R^a^*	AGTGAAGGGCTAGTGCTGTT	
LL8F^b^*	AACAGCACTAGCCCTTCACT	
LL9R^b^*	GATACACTAGGAGCAGCAGA	
LL10F^c^*	TCTGCTGCTCCTAGTGTATC	
4210ThrFWD^a^	TCGAGACTGATAAGATGGGG	Evaluation of *CoALS4210* allelic variability ^a,b,c^ indicate primer pairs used for PCR analysis represented in Fig 4 ^a^*^,b^*^,c^* indicate primer pairs used for PCR analysis represented in Fig 5
4210ThrREV^a^	AGGCTAGTAGGAGTAATTGG	
4210StalkFWD^b,a^*	CCAATTACTCCTACTAGCCT	
4210ProbeREV^b,c^*	CTCAGTTGCCAAGTGTGAAG	
LL3R^a^*	CTGTGTAGGGCTGGTACTAT	
LL4F^b^*	ATAGTACCAGCCCTACACAG	
LL5R^b^*	GTGTGGAAGTGTAGCTGAAG	
LL6F^c^*	CTTCAGCTACACTTCCACAC	
4220ThrFWD^a^	TTACATGGTCACCTTACAGC	Evaluation of *CoALS4220* allelic variability ^a,b,c^ indicate primer pairs used for PCR analysis represented in Fig 4 ^a^*^,b^*^,c^* indicate primer pairs used for PCR analysis represented in Fig 5
4220ThrREV^a^	CTGTCCAGAATGTGGTCAAC	
UpstreamTR^b^	GTTGACCACATTCTGGACAG	
4220StalkFWD^c,a^*	AAGTACCATCTGACACGTCC	
4220Stalk REV^b,c,b^*	CCATGTAGCGAATTAACCGAAG	
LL1R^a^*	CCGTATGACGCATAAGTAGATC	
LL2F^b^*	GATCTACTTATGCGTCATACGG	
800RtF2	GTGTGCTGGAGATTCGTTTC	Analysis of *CoALS* gene expression
800RtR2	ACTTCATTACCGTTGGCACC	
4210RtF	AGACCCCACTAGCCACTTCT	
4210RtR	GCCTGATCCATTTCCACCATT	
4220RtF	CATGGTGGACATTGTCACAAC	
4220RtR	GCCCGAACCTTCATAAGTGT	
OrthoACT1RtF	TTCCCAGGTATTGCTGAACG	
OrthoACT1RtR	GGAAAGTGGACAA TGAAGCC	

### Quantitation of relative gene expression levels

Relative expression of the *CoALS* genes was determined by real-time reverse transcription (RT)-PCR starting from total RNA of *C*. *orthopsilosis* isolates. Each strain was inoculated in 10 ml of YPD and grown for 16 h at 30°C with shaking. An aliquot (500 μl) of the pre-inoculum was then inoculated in 20 ml of fresh YPD broth and incubated for 1 h and 24 h at 30°C. Total RNA was extracted using Nucleospin RNA (Macherey Nagel, Düren, Germany) according to manufacturer’s instructions and treated with DNase (Macherey Nagel) to remove DNA contamination. RNA was eluted in 60 μl of RNase-free water and stored at -80°C. The quality and quantity of the extracted RNA were determined spectrophotometrically in an UVette 220–1600 (10 mm path length, 100 ml of sample volume, Eppendorf, Milan, Italy). One μg of total RNA in a 20-μl reaction volume was converted into cDNA with random primers, using the Reverse Transcription System kit (Promega), following manufacturer’s instructions. An RT-negative control was included to ensure lack of genomic DNA contamination.

Primer sequences for real-time PCR are shown in Table 1. Each PCR mixture (20 μl) contained 1 μl of cDNA, 10 μl of Sso Advanced universal SYBR Green supermix, 1 μl each of primers (final concentration 0.2 μM) and 7 μl of sterile MilliQ water. Real-time PCR was performed in 96-well plates on CFX96 Touch Real-Time PCR Detection System (BioRad) (95°C incubation for 60 s, followed by 40 cycles of 95°C incubation for 5 s and 58°C for 15 s). *C*. *orthopsilosis ACT1* was used as the reference gene (Table 1). The transcription level of *ALS* genes was calculated using the 2^- ΔCt^ method [29]. RT-PCR results were evaluated by Repeated Measures ANOVA test, followed by Dunnett’s Multiple Comparison Test. A *P* value <0.05 was considered statistically significant.

## Results

### Identification and DNA sequence of *C*. *orthopsilosis ALS* genes

**Fig 1:**
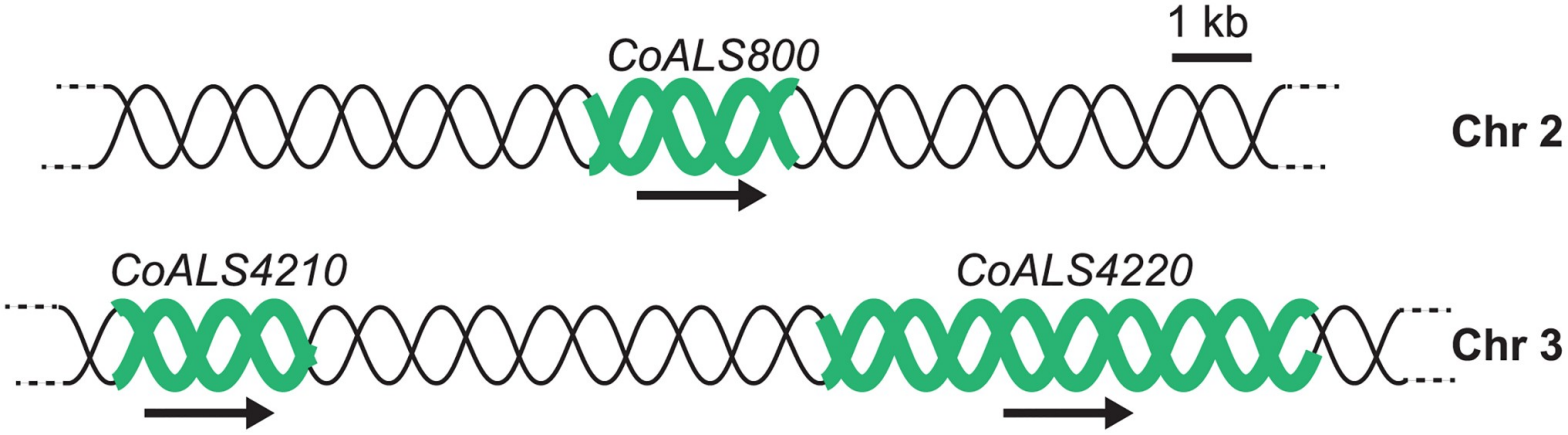
Schematic of *CoALS* genes. Analysis reported here indicated that *C*. *orthopsilosis* strain 90–125 encoded three *ALS* genes (in green), namely *CoALS800* (2499 bp) located on chromosome 2, and *CoALS4210* and *CoALS4220* (2457 bp and 6078 bp, respectively), which were contiguous on chromosome 3 and separated by 6321 nt. The scheme was drawn to scale. An arrow below each gene indicates the orientation of the *ORF*.

The *C*. *orthopsilosis* strain 90–125 genome sequence initially was accessed using CGOB3 (http://cgob3.ucd.ie; [27, 28]) and three putative *ALS* genes were located. Subsequently, data available at http://ncbi.nlm.nih.gov/genome/12421 were used to more carefully describe the *ALS* genes in the reference genome assembly (ASM31587v1). One *ALS* gene was located on chromosome 2 (*CORT_0B00800*) and two more in tandem on chromosome 3 (*CORT_0C04210* and *CORT_0C04220*; Fig 1). For simplicity, the gene names were abbreviated here as *CoALS800*, *CoALS4210*, and *CoALS4220*, respectively.

*In silico* analysis revealed that the sequences of *CoALS800* and *CoALS4220* were incomplete due to mis-assembly of repeated DNA sequences in the coding region (Table 2).

**Table 2:** Comparison between *ALS* genes in *C*. *orthopsilosis* strain 90–125 genome assemblies ASM31587v1 and PQBP00000000.

**Assembly ASM31587v1**			
**Gene**	***CoALS800***	***CoALS4210***	***CoALS4220***
**Chromosome**	2	3	3
**Chr. Sequence**	HE681720.1	HE681721.1	HE681721.1
**Protein ID**	CCG21801.1	CCG25794.1	CCG25795.1
**Coordinates**	153929..155230	953854..956106	962425..967092
**Description of encoded protein**	Encodes 432 aa of the N-terminal end; sequence truncated in the SSSEPP repeats; spliced to C-terminal end with added “nnn”	Encodes a 750-aa protein; annotated as Mms21	Encodes N-terminal 733 aa and C-terminal 750 aa; spliced together with “nnn”; length unknown
**Assembly PQBP00000000**			
**Gene**	***CoALS800***	***CoALS4210***	***CoALS4220***
**Contig**	1	5	5
**Coordinates**	2284515..2282017	959,854..962,310	958,631..974,718
**Description of encoded protein**	Encodes a complete 832-aa protein	Encodes a complete 818-aa protein	Frameshifts present; corrected by Sanger sequencing

The genome assembly was generated from short-read sequences (454 Life Sciences and Illumina) with the aid of paired-end Sanger sequence reads from a fosmid library [13]. Because fungal species tend to encode multiple *ALS* genes, each containing long stretches of repeated DNA, *ALS* genes are very difficult to assemble from short-read sequence data. The recent development of long-read DNA sequencing methodology provided the potential to produce sequence reads that span entire repeat regions. One drawback of the long-read technology is reduced accuracy of base calling [30], so Illumina data were also generated and incorporated into the genome assembly. The assembled genome was deposited in GenBank with the accession number PQBP00000000. The genome assembled into 10 contigs that mapped to the 8 chromosomal sequences defined by the reference genome assembly (ASM31587v1; Table 3). Long-read sequence data contributed to an improved assembly. For example, assembly ASM31587v1 had 242 contigs in 8 scaffolds, an N50 of 120 kb, and an L50 of 36. Assembly PQBP00000000 had no added Ns, an N50 of 1.59 Mb, and an L50 of 4.

**Table 3:** Comparison between chromosomes/contigs from the ASM31587v1 (454/Illumina) and PQBP00000000 (ONP/Illumina) assemblies.

Assembly ASM31587v1	Size (Mb)	Assembly PQBP00000000	Size (Mb)
Chr 1	2.94	Contig 3	1.62
		Contig 11	1.30
Chr 2	2.43	Contig 1	2.44
Chr 3	1.64	Contig 5	1.66
Chr 4	1.59	Contig 7	1.59
Chr 5	1.47	Contig 9	1.49
Chr 6	1.03	Contig 13	1.03
Chr 7	0.94	Contig 54	0.61
		Contig 56	0.36
Chr 8	0.61	Contig 16	0.62

The new genome assembly was searched using the Basic Local Alignment Search Tool (BLAST; https://blast.ncbi.nlm.nih.gov/Blast.cgi) with *C*. *albicans* Als3 (CaAls3) as the query (translated from GenBank accession number AY223552). BLAST results revealed the same three genes discussed above (*CoALS4210*, *CoALS4220*, *CoALS800*). Additional BLAST, using the *CoALS* sequences and other parts of known *ALS* genes as queries, failed to reveal additional genes suggesting that strain 90–125 encoded three *ALS* genes. The schematic of the chromosomal arrangement of the *C*. *orthopsilosis ALS* genes (Fig 1) accurately depicts both genome assemblies. Final sequences for the *CoALS* genes were deposited in GenBank under accession numbers MG799557 (*CoALS800*, 2499 bp), MG799558 (*CoALS4210*, 2457 bp), and MG799559 (*CoALS4220*, 6078 bp).

The information above was the most concise description of the path toward identifying the *CoALS* genes and validating their DNA sequences. Prior to generating the new genome assembly *CoALS* sequence assembly was attempted by subcloning and PCR amplification of various gene fragments, and Sanger sequencing of the resulting constructs and products. Other GenBank deposits of strain 90–125 sequences were made during the course of the study and listed here for the sake of completeness. These included KJ679579 (which was identical to MG799557), KX961387 (a partial sequence including the 5’ domain of *CoALS4210*, which was 100% identical to MG799558 in the region of overlap), and KY211672 (a partial *CoALS4220* sequence, which was assembled using Xs to indicate unknown nucleotides within the tandem repeat region).

Comparisons between data from the different approaches suggested only minor differences. For example, *CoALS4210* was predicted to be shorter in the ASM31587v1 assembly than the PQBP00000000 assembly. Validation methods pointed to the MG799558 sequence as the correct, final version. For *CoALS4220*, the long-read sequence technology provided an accurately sized template for assembly of the tandem repeat sequences. Sanger sequencing of subcloned fragments and PCR products in the different laboratories contributing to this manuscript were in agreement with the exception of 6 nucleotides in tandem repeat unit 14 (TR14); the shorter version was reported in MG799559 and featured in this manuscript. The 90–125 isolate used in all work originated in the Tavanti laboratory.

### Features of *C*. *orthopsilosis* Als proteins

**Fig 2:**
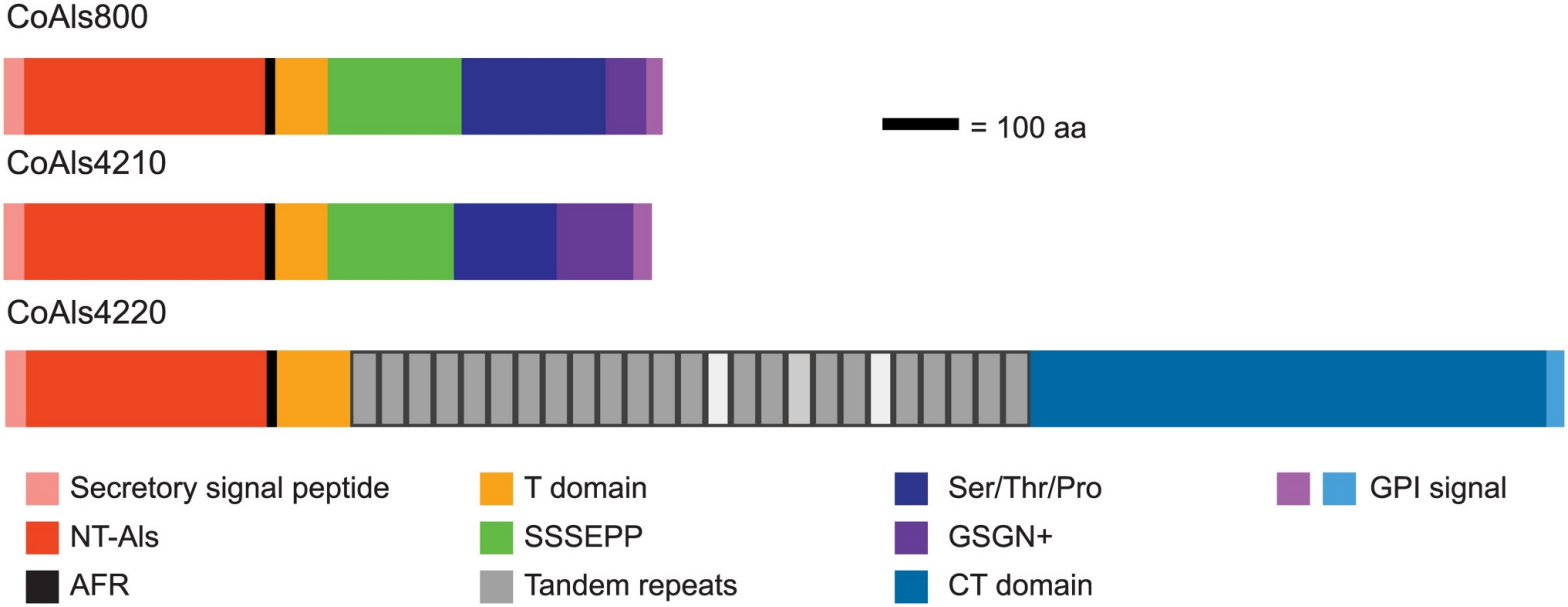
Domain architecture of CoAls proteins. The three CoAls protein sequences were drawn to scale, with domains represented by different colors. Each protein encoded a secretory signal peptide followed by the N-terminal domain (NT-Als) where adhesive function resides in the well-characterized *C*. *albicans* orthologs [4, 7]. Each CoAls protein also had a short amyloid forming region (AFR; [8]), and a Thr-rich region (T) domain. Like Als proteins in *C*. *albicans*[2], CoAls4220 included a central region of conserved tandemly repeated sequences. Most units were 36 aa, but two (TR14 and TR20) had 34 aa and one (TR17) had 35 aa. CoAls4210 and CoAls800 lacked the central tandem-repeat region. Instead, they had a short, imperfect repeated sequence (SSSEPP consensus), a Ser/Thr/Pro-rich region, and another short, imperfect repeat (GSGN consensus). Each CoAls protein had C-terminal signal for GPI anchor addition, predicting its localization in the fungal cell wall [3].

**Fig 3:**
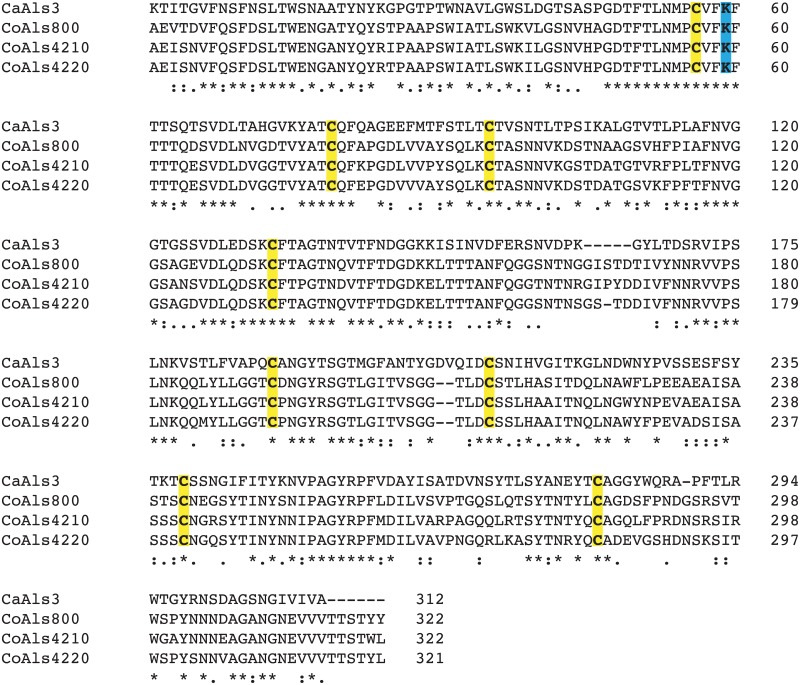
Conserved NT-Als features suggested adhesive activity for CoAls proteins. The mature (signal peptide removed) NT domains of CoAls800, CoAls4210, and CoAls4220 contained 8 Cys residues in conserved positions (highlighted in yellow), which are essential for the folding of *C*. *albicans* (Ca) NT-Als3 for which the three-dimensional structure was solved [7]. Conservation of NT-Als3 adhesive function in the CoAls proteins was also suggested by the presence of the invariant Lys (K59) located in the CaNT-Als3 binding cavity (highlighted in blue). The amino acid alignment was produced using Clustal Omega (https://www.ebi.ac.uk/Tools/msa/clustalo). Identical (*), conserved (:), and semi-conserved (.) amino acids are indicated below the alignment. Dashes in the sequence indicate gaps. The sequence of *C*. *albicans* Als3 (CaAls3; GenBank accession number AY223552) was used as a reference.

*C*. *orthopsilosis ALS* genes were translated to visualize and compare the CoAls proteins (Fig 2). Protein features were compared to the well-characterized *C*. *albicans* proteins [2]. Each CoAls protein encoded a secretory signal sequence of 22 amino acids followed by an N-terminal (NT) domain of 312 or 313 amino acids. The CoAls NT domains were 81–87% identical, and shared 45–47% identity with NT-Als3 from *C*. *albicans*. Alignment of the NT-CoAls amino acid sequences with NT-Als3 for which the three-dimensional structure is known [7] showed conservation of the eight Cys that provide the NT-Als3 fold (Fig 3). This sequence similarity suggested conservation of adhesive function in the CoAls proteins. The NT domain was followed by a short sequence (AFR) that had amyloid-forming potential as defined by Tango [24]. The aggregative function of this sequence was demonstrated previously in *C*. *albicans* Als proteins [7, 8].

Like CaAls3, the CoAls proteins had a Thr-rich region (T domain; 32–34% Thr) that followed the NT and AFR sequences. The boundaries of the T domain were based on evaluations of sequence data, rather than on functional data. Currently the T domain is bounded in *C*. *albicans* Als proteins by the end of the AFR and the start of the tandemly repeated sequences [7]. Of the newly described CoAls proteins, only CoAls4220 had tandemly repeated copies of a 36-aa sequence. Unlike *C*. *albicans* Als proteins, however, the length of selected repeat units in CoAls4220 was variable, with some repeat units lacking one or two amino acids. The region C-terminal to the tandem repeats in CoAls4220 was rich in Ser (30%) and Thr (15%) similar to C-terminal regions in *C*. *albicans* Als proteins.

Regions following the T domain in CoAls800 and CoAls4210 were different than those observed in other Als proteins. Compositions of the two proteins were very similar in this region (Fig 2). Both encoded two different short, imperfect repeated sequences. The motif SSSEPP was found in the region proximal to the T domain. Following a Ser/Thr/Pro-rich (58–62%) region, a GSGN motif was present. Each CoAls protein had a C-terminal sequence with hallmarks of a GPI anchor addition site. The C-terminal 20 aa were predicted to be cleaved in this process.

### Allelic variation in *C*. *orthopsilosis ALS* genes

*C*. *albicans ALS* genes are marked by considerable variation that exists between strains and between alleles in the diploid species [31, 32]. Allelic variation is notable in small nucleotide sequence changes, as well as large differences in gene length, mainly due to expansion and contraction of repeated sequences within the coding region. These observations provide the foundation for evaluation of allelic variation in the *CoALS* genes.

PCR primers were designed to amplify and sequence the 5’ end of each *CoALS* gene in the strains used in the study (Table 1). Resulting sequences were deposited in GenBank. For *CoALS800*, accession numbers included KM506766 (ATCC 96139), KM506767 (85), KJ855317 (124), KJ855318 (488), and KJ855319 (331). *CoALS4210* accession numbers were KX961388 (ATCC 96139), KX961391 (85), KX961389 (124), KX961390 (488), and KX961392 (331). *CoALS4220* accession numbers included KX961393 (ATCC 96139), KX961394 (85), KX961395 (124), KX961396 (488), and KX961397 (331). Translation of these nucleotide sequences provided 396 aa from the N-terminal end of CoAls800, 340 aa from CoAls4210, and 337 aa from CoAls4220, including the signal peptide for each protein. Alignment of all sequences for the same protein showed >98% identity, suggesting little variation in the adhesive domain across strains. None of the altered amino acids in any of the proteins was located within the peptide-binding cavity.

**Fig 4:**
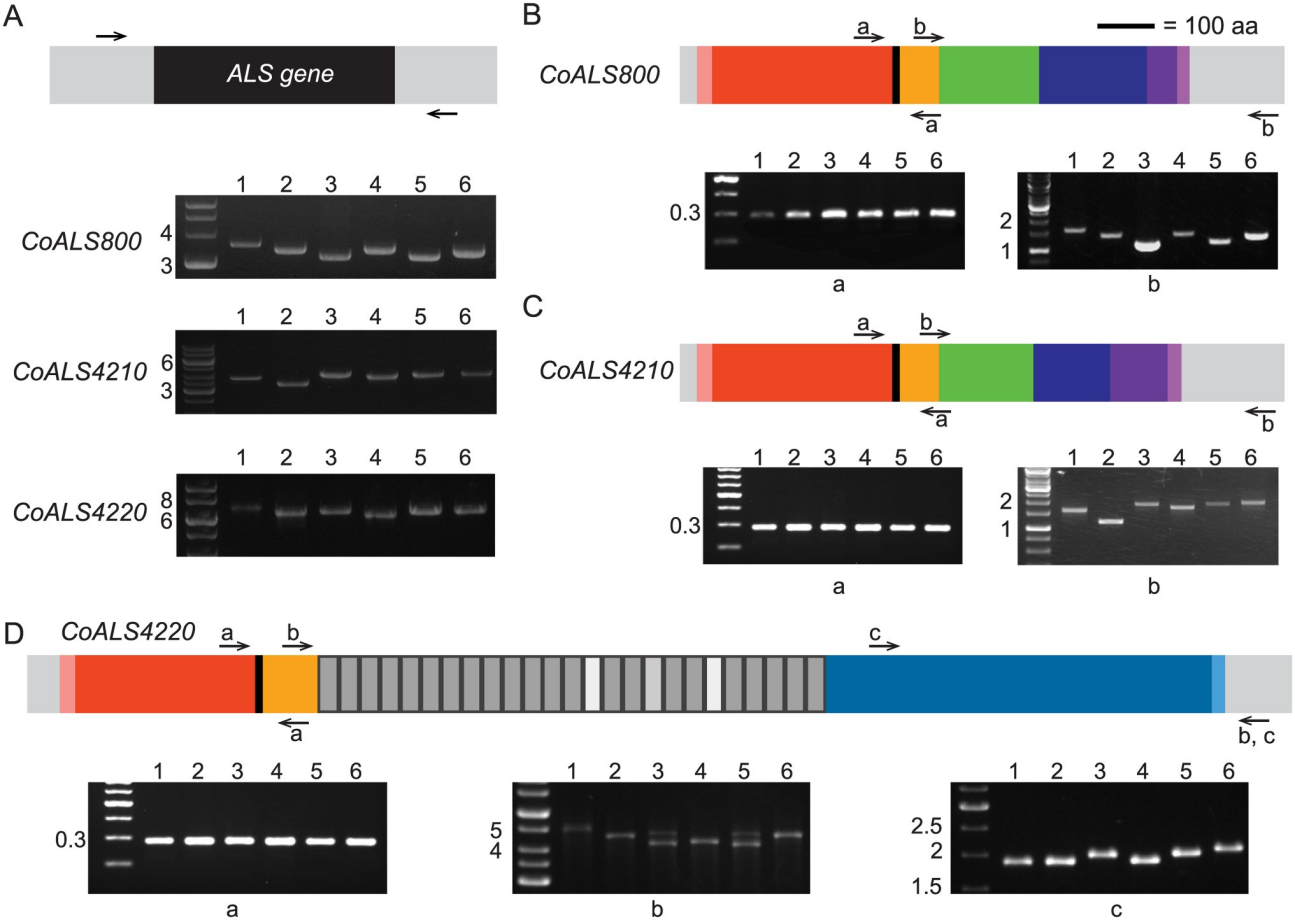
Source of allelic variability in *CoALS* genes and their encoded proteins. A PCR-based strategy was used to evaluate the presence of allelic variation among *CoALS* genes from *C*. *orthopsilosis* strains 90–125 (1); ATCC 96139 (2); 85 (3), 124 (4), 488 (5), and 331 (6). Each subfigure shows a schematic of the *CoALS* gene or its encoded protein and corresponding PCR products that were analyzed on ethidium-bromide-stained agarose gels. Flanking gray rectangles represent the position of PCR primers outside of the coding region. (A). Overall size differences of the *CoALS* genes in each strain were demonstrated using primers 5’ and 3’ of the coding region (depicted as arrows; primer sequences are detailed in Table 1). Size markers (in kb) are indicated on the left of each gel image. Experiments used either GeneRuler 1 kb DNA ladder (Thermo Fisher Scientific) or 100 bp DNA ladder (NEB). Dissection of the source of the allelic variation in genes *CoALS800* (B), *CoALS4210* (C), and *CoALS4220* (D) indicated variability in the sequences encoding the C-terminal regions of each protein and the tandem repeats in *CoALS4220*. Primers are labeled with lowercase letters that correspond to the labels on the agarose gel images. Sizes of fragments encoding the AFR and T domains were not detectably different between strains.

PCR was used to assess length variation among the *CoALS* genes. Length variation was apparent from amplification of the entire *CoALS* coding region (Fig 4A). Targeted PCR primers were designed to attribute this length variation to specific regions of each gene. Length variation was present in sequences 3’ of the AFR-encoding region (Fig 4B, 4C and 4D). Variation between the diploid alleles was obvious in some strains within the *CoALS4220* tandem repeat domain (Fig 4D).

**Fig 5:**
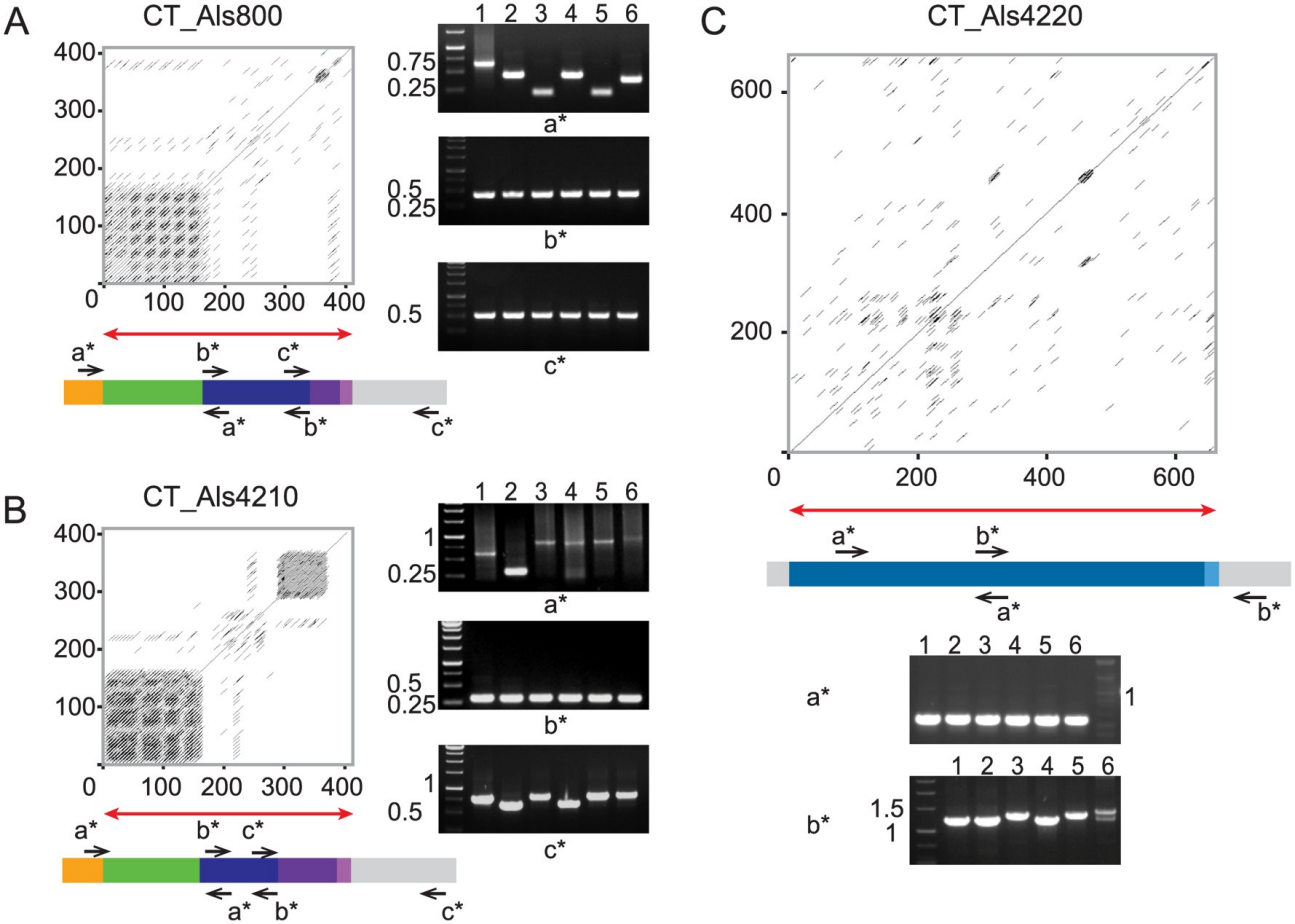
Dissection of allelic variability in CT-encoding regions of *CoALS* genes. Each panel contains the schematic of a CoAls protein (A = CoAls800; B = CoAls4210; C = CoAls4220) carried forward from Figs 2 and 5, agarose gel images that reveal PCR product sizes from amplification with different primer pairs (labeled in lowercase letters), and a Dotmatcher output (http://www.bioinformatics.nl/cgi-bin/emboss/dotmatcher) that compares each amino acid sequence to itself to reveal repeated sequences. The analyzed region is indicated by a red arrow in each panel. Strain numbers are the same as for Fig 4. Primer sequences are shown in Table 1. Molecular sizes (in kb) are shown at the left of each gel image. GeneRuler 1 kb DNA ladder (Thermo Fisher Scientific) was used in all experiments. Variability in the CT region of CoAls800 was located in the SSSEPP region (A), while in CoAls4210, the GSGN region was also variable in size. The CT region of CoAls4220 was also variable in size, due to sequence differences that encode the 3’ half of the CT region (C).

Additional primers were designed to further dissect the location of the observed length variation (Fig 5). Strain and/or allelic variability was noted in the SSSEPP-encoding sequences of *CoALS800* and *CoALS4210*. The GSGN-encoding sequences in these two genes were homogeneous in *CoALS800*, but variable in *CoALS4210*. Variability was observed in the 3’ end of the CT-encoding domain of *CoALS4220*. These sequence differences suggest that mature CoAls proteins will be different sizes across strains, and that within a strain, alleles may produce proteins of different lengths.

### Real-time PCR analysis of *C*. *orthopsilosis ALS* gene expression

**Fig 6:**
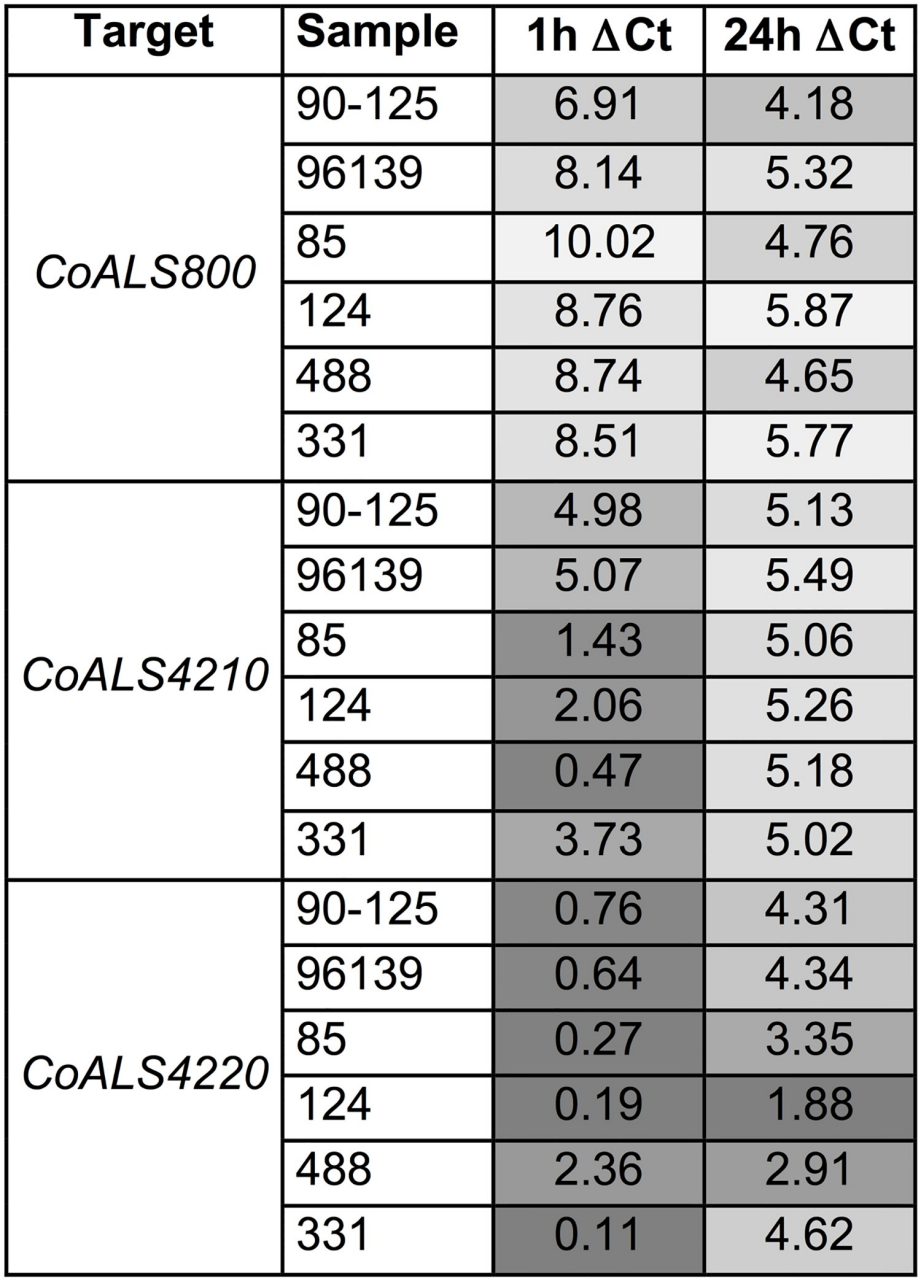
Strain- and growth-stage differences in *CoALS* gene expression. Real-time RT-PCR was used to quantify relative expression levels for the three *CoALS* genes in the six *C*. *orthopsilosis* strains grown for either 1 h or 24 h at 30°C in YPD liquid medium. Lower numbers indicate a smaller difference between expression of the gene and the *ACT1* control, suggesting higher overall relative expression. Gray-scale coding indicates higher (darker gray) and lower expression (lighter shading). *CoALS800* showed the lowest expression level at 1 h incubation in all the strains tested (*P*<0.0001). *CoALS4220* was expressed more highly than the other genes (*P*<0.0001 at 1h, *P*<0.001 at 24h), although its transcription level was lower at 24 h compared to 1 h.

Quantitative expression of *CoALS* genes was measured in the clinical isolates and reference strains grown in YPD medium for 1 h and 24 h. Data were displayed as a heat map (Fig 6). Transcription levels for the three *CoALS* genes varied based on stage of growth. *CoALS800* showed the lowest expression level at 1 h incubation in all the strains tested (*P*< 0.0001). Conversely, *CoALS4220* was expressed more highly than the other two genes (*P* < 0.0001 at 1 h, P < 0.001 at 24 h), although its transcriptional level was lower at 24 h compared to 1 h. Strain differences in expression were observed for all *CoALS* genes.

## Discussion

The study of microbial pathogenesis has been revolutionized by the availability of genome sequences for many species. Although sequence data can be generated and assembled into draft genome files at a rapid pace, some genes have features that defy accurate representation in these resources. Examples include genes that belong to families with many similar loci, and open reading frames that encode multiple copies of repetitive sequences. Genes in the *ALS* family possess both features, and as such, are often mis-assembled in available genome sequences.

Long-read DNA sequence methodology is one answer to this problem. Although long-read methods produce data with a lower accuracy in base calling [30], the method is attractive for studying the *ALS* family since the long-read sequence can provide a template upon which shorter-read data (i.e. Illumina) can be assembled. Work presented here demonstrates the utility of this approach. The combination of methods provided an accurate and complete assembly for two of the three *CoALS* genes. Data for the third gene was sufficiently complete that primers could be designed for PCR amplification and Sanger sequencing of the product, delivering a final gene sequence. Overall, combining long- and short-read approaches generated a more-complete picture of the *ALS* family than was evident in the previous genome sequence that was assembled without the benefit of the long-read data.

Among the newly characterized *CoALS* genes, *CoALS4220* looks most like the *ALS* genes that were described in *C*. *albicans* because of the presence of multiple copies of a tandemly repeated sequence in the center of the gene. In *CoALS4220*, however, this sequence includes repeat copies that are missing 1 or 2 amino acids, a feature that was not observed in any of the *C*. *albicans* proteins. The *CoALS800* and *CoALS4210* genes are unique among currently characterized *ALS* genes because they do not possess a tandemly repeated sequence and, therefore, are shorter than most Als proteins for which adhesive function has been demonstrated. For example, *C*. *albicans* Als3 is produced from two alleles in strain SC5314: one protein is 1155 amino acids and the other is 1047 amino acids, due to the presence of three fewer copies of the tandemly repeated sequence. The shorter protein contributes less than the larger protein to *C*. *albicans* adhesion, presumably because the longer protein is better able to project the NT-Als adhesive domain away from the *C*. *albicans* cell surface [33]. These CaAls3 sequences are over 300 amino acids longer than the CoAls800 and CoAls4210 sequences described here. However, recent work shows that *CoALS4210* contributes to *C*. *orthopsilosis* adhesion because deletion of the gene results in reduced adhesion to HBECs [34].

The current study is also unique in that it examines *ALS* gene expression in multiple clinical isolates using a quantitative method and demonstrates notable strain-specific gene expression patterns. Overall, *C*. *orthopsilosis* shows differential regulation of its *ALS* genes by growth stage, a theme that was also found in *C*. *albicans*. *C*. *albicans ALS4* is up-regulated in cells from a saturated culture [35] whereas *C*. *albicans ALS1* is highly expressed in cells that are transferred to fresh growth medium [36]. In *C*. *orthopsilsosis*, *CoALS800* was more highly expressed as a culture aged, while *CoALS4220* was more highly expressed in a 1-h culture. *CoALS4210* expression patterns varied by strain, with some strains showing higher relative expression in a young culture and others exhibiting little gene expression difference regardless of which growth stage was examined.

Future studies will be aimed at associating gene expression data with adhesive function in different experimental models. Previously characterized strain 124, described as highly adhesive to expholiated buccal cells [11], shows a strong relative expression of *CoALS4220* (Fig 6), which could be responsible for its higher relative adhesion [11], as also demonstrated in *C*. *albicans*, whose *ALS* gene expression levels are positively correlated with Als protein abundance.

*C*. *orthopsilosis* is closely related to *Candida parapsilosis*; it has been less than 15 years since the species were recognized as distinct [16]. Publications describe *C*. *parapsilosis ALS* gene content as highly divergent by strain. Pryszcz et al. [14] examined whole genome sequences from a variety of *C*. *parapsilosis* isolates and noted one strain with 5 genes, while others encoded only 1. In our current study, PCR primers designed to recognize *CoALS800*, *CoALS4210* and *CoALS4220* amplified the predicted products from each of 6 *C*. *orthopsilosis* isolates. Amplified Fragment Length Polymorphism analysis of four of the isolates (ATCC 96139, 85, 124, and 331) was reported previously [37]. UPGMA analysis showed that these strains belong to different clusters, indicating genetic diversity among the isolates used in the current study. These observations suggest broad conservation of the three *CoALS* genes within the species. Recently, it has been shown that the majority of *C*. *orthopsilosis* strains are hybrids between a Parental Species A (non-hybrid, of which the homozygous isolate 90–125 is representative) and a Parental Species B, which has not been isolated in non-hybrid form [38, 39]. Interestingly, three of the 4 clinical isolates used in this study are known to be hybrids belonging to different clades, namely strains Co85 (Clade 1), Co331 (Clade 2), and Co124 (Clade 4.1) [39]. It has been suggested that *C*. *orthopsilosis* and *C*. *metapsilosis* hybrid formation may have facilitated a change in pathogenicity to humans [39, 40]. Further analyses will be required to investigate potential association between adhesion ability and hybrid genomes.

Although Co85, Co124, Co331 and 90–125 all have diploid genomes, evaluation of copy number variation in 1-kb windows across the genomes evidenced the presence of a single copy of *CoALS4210* in strain Co85 [39]. This result did not seem to affect *ALS* mRNA levels detected by RT-PCR in strain Co85. We can conclude with certainty that strain 90–125 does not have additional *ALS* genes, but we cannot exclude the presence of other *ALS* genes in the remaining strains. Ongoing work in *C*. *orthopsilosis* will continue to characterize the *ALS* family and adhesive function of the Als proteins in this species.
